# Giant Cell Tumor of the Patella: A Case Report

**DOI:** 10.7759/cureus.68590

**Published:** 2024-09-03

**Authors:** Shivshankar Jadhav, Yogita S Rathod, Surekha R Rathod

**Affiliations:** 1 Department of Orthopedic Surgery, Datta Meghe Institute of Higher Education and Research, Wardha, IND; 2 Department of Pathology, D. Y. Patil Medical College, Kolhapur, IND; 3 Department of Periodontics and Implantology, Ranjeet Deshmukh Dental College and Research Centre, Nagpur, IND

**Keywords:** giant cell tumor of the patella, orthopedics, histopathology (hp), diagnostic radiology, oncology

## Abstract

Giant cell tumors (GCT) are uncommon as primary tumors localized within the patella. This is a case report of a 25-year-old male who developed a GCT in his patella. The patient had intermittent right anterior knee discomfort for a year before presentation. The radiological features pointed to a benign illness. The GCT of the bone was the intraoperative pathological diagnosis. Radiation curettage and adjuvant therapy consisting of phenol and ethanol injections and calcium phosphate cement were used to treat the lesion. Histologically, the tumor comprised several large osteoclastic cells mixed in with spherical- or spindle-shaped mononuclear cells. Sixteen months following surgery, the patient had no symptoms and no signs of distant metastasis or local recurrence. In particular, in young individuals, patellar GCTs may be included in the differential diagnosis of anterior knee discomfort and/or edema despite their rarity.

## Introduction

Patellar tumors are uncommon as the patella is a cancellous bone and often has a benign appearance instead of a malignant one. According to statistics, 73% of patellar tumors are benign. This represents the bulk of the tumors. Giant cell tumors (GCTs) and chondroblastomas, which make up 33% and 16% of cases, respectively, are included in the benign category [1]. Four to five percent of all primary bone lesions are GCTs of the bone (GCTBs). Even though patellar bone involvement accounts for less than 1% of all GCTB cases, GCT is a very aggressive localized condition that generally affects women more often than men and typically affects young individuals between the ages of 20 and 45 [2,3]. According to a literature study, GCT often affects the sacrum, spine, and epiphyseal area of the long bones [4]. Reporting a rare case like GCTB is motivated by the statistics associated with its diagnosis. GCTs are extremely uncommon; just one in 1,000,000 people get them annually, and 50% of those cases are localized at the knee joint [5]. Patients usually appear with anterior knee discomfort, joint swelling, and intensifying pain during rest despite the condition's rarity and predominance in the knee joints [6]. The diagnosis of patellar tendinitis, chondroblastoma, and aneurysmal bone cyst (ABC) is more prevalent than that of GCT, so the latter is frequently diagnosed later.

On the other hand, noninvasive investigations like ultrasound, magnetic resonance imaging (MRI), CT, and plain radiographs followed by biopsy can be done when suspecting a case of GCT. This can be followed by broad excision of the tumor or curettage during surgery. Denosumab, a targeted medication that targets the receptor activator of the nuclear factor kappa-Β ligand molecule, is being studied further to see whether it can control the growth of GCTs. It is frequently used before surgery to help patients with the condition recover better after surgery [6].

## Case presentation

A 25-year-old male with a one-year history of sporadic right anterior knee discomfort in the form of pain without any restriction of knee movements reported to our hospital. The patient had stumbled and was experiencing increasing anterior knee discomfort for two weeks before arriving at our hospital. Upon physical examination, the right knee's anterior side showed minor discomfort in the form of pain and slight edema. The edema was present anterior to the patella. Nevertheless, we did not notice any redness, skin adhesion, localized heat, or restriction of the knee's range of motion. Moreover, there was no evidence of joint effusion. All laboratory results were within normal ranges, except for a slight increase in alkaline phosphatase levels of 180 international units per liter (IU/L) (normally, it ranges from 44-147 IU/L). The patient had an unremarkable prior medical history.

**Figure 1:**
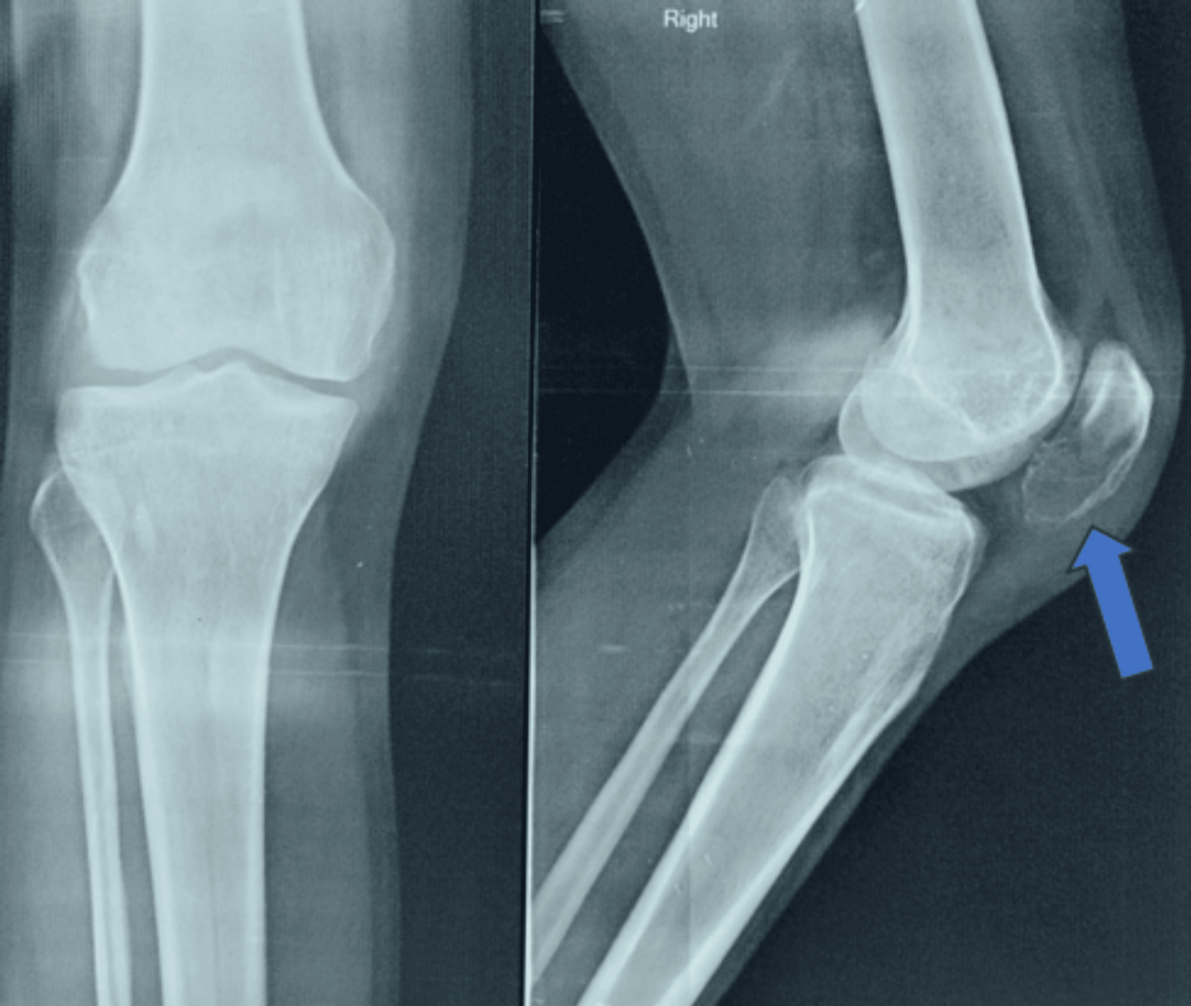
Anteroposterior and lateral views of the knee joint, showing lytic lesion in the patella. The blue arrow shows the lytic lesion with a thinned cortex in the inferior region of the patella

**Figure 2:**
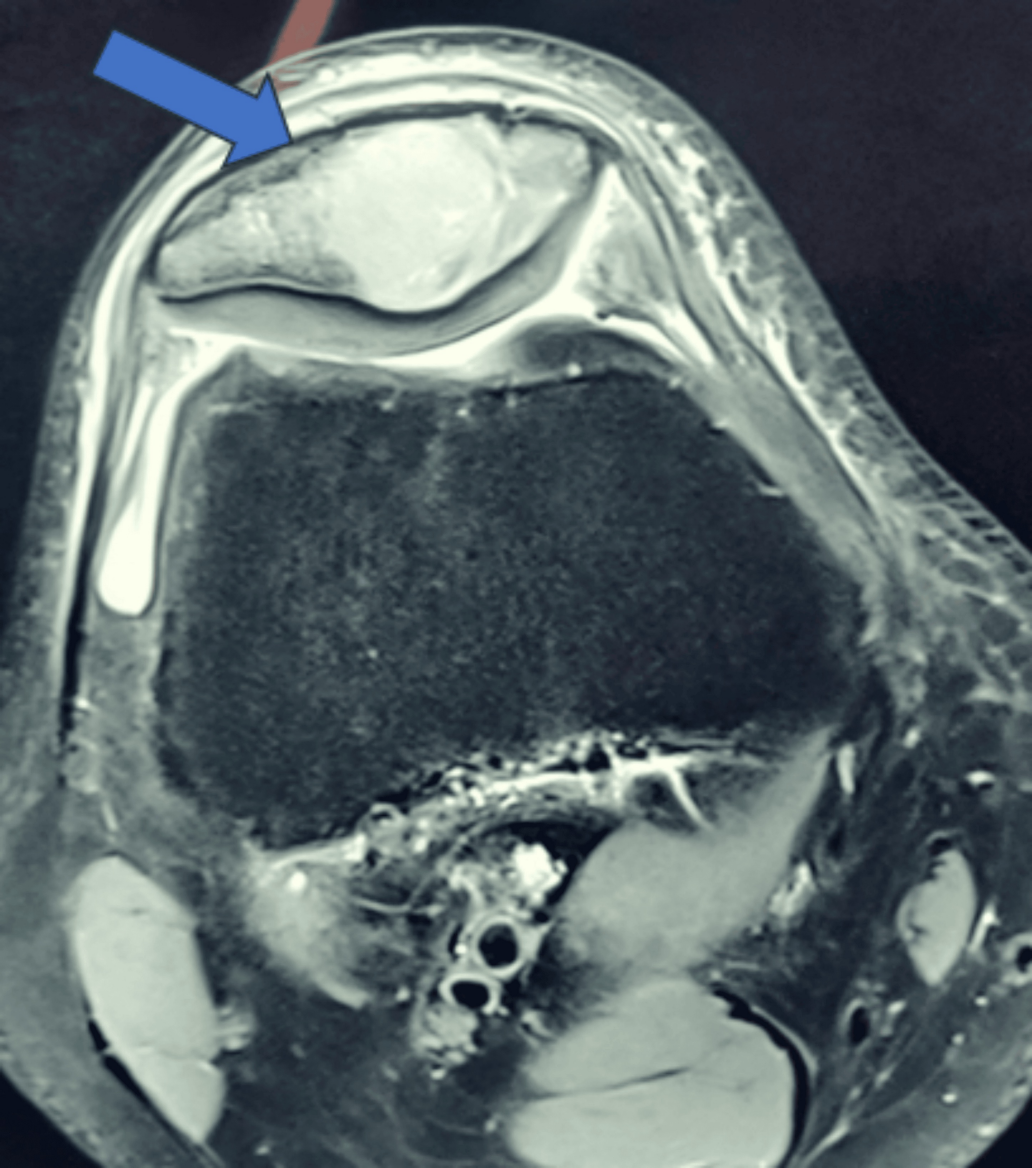
Heterogeneous signal intensity on the T2‑weighted MRI scan, showing heterogeneous signal intensity with an intraosseous lesion in the patella (blue arrow) MRI: magnetic resonance imaging

A distinct lytic lesion was visible on plain radiographs, located in the inferior and central regions of the patella (Figure 1). The periosteal reaction was not seen. The lesion showed heterogeneous signal intensity on T2-weighted MRI scans (Figure 2). There was no discernible intra-articular or soft-tissue extension.

Following an incisional biopsy, GCTB was an intraoperative pathological diagnosis. After that, the patient had radical intralesional curettage with a high-speed burr. After curettage, a cotton swab was used to carefully apply 70% phenol to the cavity's interior surface for five minutes. Then, 99.5% ethanol was poured into the cavity for five minutes. Following a thorough cleaning with a standard saline solution, calcium phosphate cement was used to fill the cavity.

**Figure 3:**
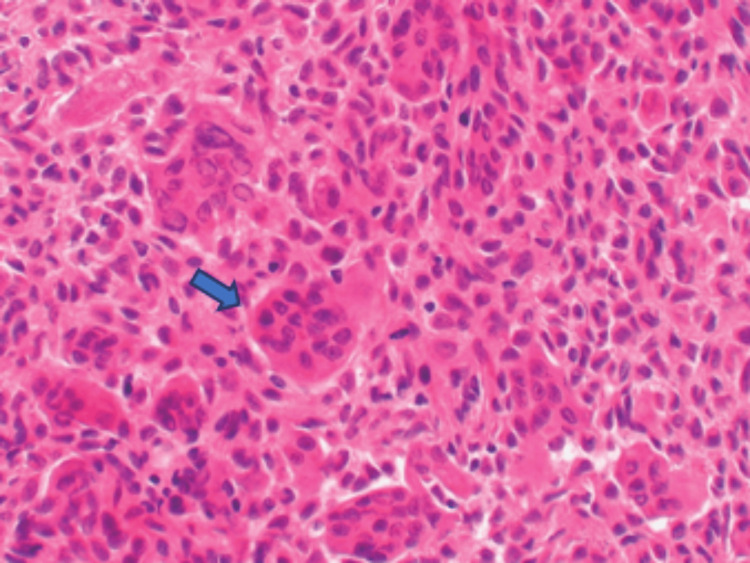
Histopathological image (H&E stain, 10× magnification), showing multiple giant cells and calcifications (blue arrow) H&E: hematoxylin and eosin

Upon microscopic examination, the tumor was found to be composed of several large osteoclastic cells interspersed with spherical- or spindle-shaped mononuclear cells. Histiocyte aggregates, hemosiderin deposits, and bleeding foci were also found (Figure 3). There were mitotic figures among the mononuclear cells, but no abnormal mitoses were seen. These results confirmed the GCTB diagnosis. The postoperative period was uneventful. After surgery, the patient resumed regular activities within a month. The patient had no symptoms at the 16-month follow-up, and there was no indication of a distant metastasis or local recurrence.

## Discussion

An uncommon benign tumor called a GCT makes up around 5% of primary bone tumors; 40%-50% of these instances are known to involve the knee joint [7]. A case by Slavchev et al. demonstrates that the knee joint is affected at a stunning incidence of 70% [2]. GCT entity is now considered a differential when evaluating any persistent knee joint pain in relation to flexion and extension against resistance or physical activity. The condition primarily affects adolescents and age groups limited to 30-40 years old [8]. Even though the tumor is benign and rarely occurs, it has been discovered to be locally aggressive [9].

The GCT of the patella begins to show symptoms such as swelling over the knee associated with pain. Additional symptoms of the condition include joint erythema, discomfort on palpation, effusion, and crepitus on physical examination. The clinician may ignore the severity of symptoms of knee pain and swelling, and the patient gets treated for pain rather than finding the cause for those symptoms. Therefore, when evaluating young individuals with prolonged knee discomfort while consuming pain suppression treatment on and off, a diagnosis needs to be made after proper investigations to rule out the differential diagnosis. Laboratory measures have demonstrated that affected patients have elevated blood levels of alkaline phosphatase and erythrocyte sedimentation rate [10].

Tumors in the patella are a very uncommon etiology of anterior knee pain, thus delaying the diagnosis. Malignant tumors are rare compared to benign lesions. GCT is the most common benign tumor, followed by chondroblastoma and ABC [11]. Chondroblastoma and hemangioma may be considered as differential diagnoses in this case. Chondroblastoma accounts for less than 1% of all tumors of bone, is male-predominant, and occurs in the second decades of life [12]. Compared to GCT, chondroblastoma is smaller in size [13]. Periosteal reaction, calcifications, and septations are absent in the patella in cases of hemangioma.

Imaging methods are also not essential for diagnosing morbidity [14]. Imaging modalities are highly recommended and most important for diagnosing morbidity in cases with GCTB since staging is very important. Plain radiography and MRI are useful in raising suspicion of the illness, which is then confirmed by biopsy. It has been declared that using radiography for an initial examination is quite beneficial in identifying morbidity. Yoshida et al. shed light on the tumor's suspected presence, which was predominantly determined by the MRI and radiography results, emphasizing the significance of early imaging modalities [15]. The two components of the surgical technique have been described as intralesional curettage and partial patellectomy, with surgical excision of the tumor being the preferred course of action. Curettage with bone graft and patellectomy are the surgical procedures used to treat patellar GCT [16]. The best course of action for aggressive benign lesions with cortical breakthroughs is total patellectomy. Malhotra et al. recently described a case of aggressive GCT of the patella with broad excision and restoration of the extensor mechanism utilizing a patellar allograft [17]. Some recent advances in the management of GCTB include the use of denosumab, bisphosphonates, chemical adjuvant therapies (alcohol and phenol), and radiotherapy. Although there is not a clear treatment regimen in the literature, publications do discuss the use of patellectomy for malignant tumors.

## Conclusions

Despite being rare, patellar tumors should always be considered a potential cause of pain and discomfort in the anterior knee, along with patellar tendinitis, chondroblastoma, and ABC. It is crucial to remember that patellar GCTs might be challenging to differentiate from other GCTBs with standard diagnostic imaging, necessitating a histological examination of the material removed after surgery. When choosing treatment choices that significantly depend on the tumor's stage, surgeons must proceed with caution.
